# Nonlinear Adaptive Control of Bipolar Mood Disorder: A New Approach for Quenching the Mood Swing

**DOI:** 10.3390/biomedicines13123090

**Published:** 2025-12-15

**Authors:** Ugur Hasirci

**Affiliations:** Electrical and Electronics Engineering Department, Duzce University, 81620 Düzce, Türkiye; ugurhasirci@duzce.edu.tr

**Keywords:** bipolar mood disorder, nonlinear control, adaptive control, numerical simulation, Lyapunov stability

## Abstract

**Background/Objectives**: Mood disorders are described by marked disruptions in emotions. Generally speaking, mood disorders are classified into two main categories: unipolar mood disorder, also known as unipolar depression, and bipolar mood disorder, also called manic-depressive illness. It is estimated that 40 million people live with bipolar disorder worldwide. Mathematical modeling of the dynamics of bipolar disorder may help to both better understand and treat the illness. This is especially important for bipolar disorder since, unlike unipolar disorder, there is an oscillation to be quenched between hypomanic and depressive episodes. **Methods**: By using a nonlinear dynamical model of bipolar disorder, this study offers two different control (treatment) approaches for the disorder. The first one is a nonlinear exact model knowledge controller assuming that all the parameters of the patient model are known. The second one is a nonlinear adaptive controller assuming that all the parameters are unknown. **Results**: Both controllers aim to drive both emotional mood and the change rate to a stable state. The Backstepping Technique is utilized as a nonlinear controller design tool. For both controllers, Lyapunov-type arguments are used to design the controller and to prove the stability of the designed controllers. Numerical simulation results are also provided to show the performance and feasibility of the proposed controllers. **Conclusions**: It has been shown that a nonlinear controller is capable of driving the emotional mood to its equilibrium point, zero, by quenching the mood swing.

## 1. Introduction

Mood is defined as a pervasive and sustained feeling tone that is endured internally and impacts nearly all aspects of a person’s behavior in the external world. Mood disorders are described by marked disruptions in emotions [1]. Generally speaking, mood disorders are classified into two main categories: unipolar mood disorder, also known as unipolar depression, and bipolar mood disorder, also called manic-depressive illness. Up-to-date statistics provided by the National Institute of Mental Health [2] reveal the rising prevalence of the disorders, which is 23.1% of U.S. adults. While unipolar depression is characterized by a persistent low mood, bipolar disorder, on the other hand, is characterized by shifts in two episodes: manic and depressive. According to the World Health Organization report [3], it is estimated that 40 million people worldwide live with bipolar disorder.

Bipolar disorder is also classified into some subclasses [4]. As a general classification, Bipolar I Disorder is characterized by a mood swing between manic and depressive episodes with rapid swinging, while Bipolar II Disorder is characterized by a mood swing between hypomanic and depressive episodes with a relatively slower swing. For example, a manic episode may last 1–2 days in Bipolar I Disorder, while hypomanic episodes may last 1–2 week or more and be more severe in Bipolar II Disorder, which is the focus of this study.

More dramatic consequences of Bipolar II Disorder motivate researchers to understand its dynamics. Mathematical modeling of its dynamics may help to both better understand and treat the illness. This is especially important for bipolar disorder since, unlike unipolar disorder, there is an oscillation to be quenched between hypomanic and depressive episodes. Efforts on the control (treatment) of bipolar disorder need a differential equation model of it since all continuous dynamic systems can be modeled by a differential equation. Many researchers have been proposed such differential equation models for Bipolar II Disorder.

**Figure 1:**
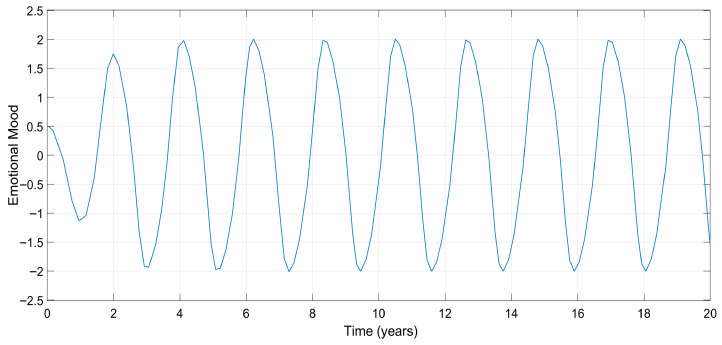
Mood versus time with $x\left(0\right)=0.5$ in the untreated case.

A clinically useful differential equation model of Bipolar II Disorder has been proposed by Daughtery et al. [5] as

(1) $$\ddot{x}(t)+b\left[a-x^{2}\left(t\right)\right]\dot{x}+cx(t)=0$$

where $x\left(t\right)$ is emotional mood, so its time derivative, $\dot{x}(t)$, represents a mood change. Note that this equation is unforced; i.e., there is no input to the system in a mathematical manner. From a physiological point of view, no term represents the treatment since the right-hand-side term is equal to zero. The solution to this differential equation tends to zero if the initial conditions are all zero; i.e., $x\left(0\right)=0$ and $\dot{x}\left(0\right)=0$. But, on the other hand, any initial condition of mood, x(0), other than zero, will lead to a fixed-amplitude oscillation at steady state. Figure 1 shows such an oscillation for $x\left(0\right)=0.5$ and for reasonable values of the systems constants, namely a=1, b=2, and c=9. From a physiological point of view, untreated initial perturbation of either a manic or depressive mood will lead a continuous mood change, which is the nature of bipolar disorder. The analysis explained here makes this model more clinically reasonable.

Figure 1 clearly reveals the importance of treatment to quench the sustained oscillation, i.e., the mood swing. The existence of an external treatment allows the system model given in Equation (1) to be expressed as

(2) $$\ddot{x}(t)+b\left[a-x^{2}\left(t\right)\right]\dot{x}+cx(t)=u(t)$$

where u(t) at the right-hand side of Equation (2) represents the control input signal to be designed or, in other words, the treatment. Clinical treatment for Bipolar II Disorder is a combination of medication and therapy. As stated in clinical reports [6], medication is composed of antidepressants and antipsychotics, which both act as mood stabilizers. Therapy, on the other hand, plays an important role to accelerate the effect of the medication.

For the model given in Equation (2), Daughtery et al. [5] designed a controller (treatment) to reduce the amplitude of oscillations given in Figure 1. Mohan [7] also designed a controller to not only reduce the amplitude but also quench the oscillation by driving x(t) to zero, that is, no mood change (oscillations) at steady state (end of treatment). But there are still oscillations in the treatment protocol proposed by Mohan [7] during the transient state (treatment). Both controllers are not analytical; i.e., the controllers are not based on models. Each controller offers a standard u(t) input by regulating the control gains. The system parameters a, b, and c are assumed to be measurable and/or known parameters, while these parameters will have different values for different patients.

This study proposes a novel controller (treatment scheme) for Bipolar II Disorder. The main novelties provided are (i) quenching the mood swing not only at steady state but also during the transient period by using a nonlinear model-based controller and (ii) stabilizing the mood even if all the model’s parameters are unknown, which is clinically more realistic.

The rest of this paper is organized as follows: Section 2 defines the control problem by presenting a state-space model of the system. Section 3 explains an exact model knowledge controller. Section 4 provides the design of an adaptive controller assuming that all the parameters are unknown. Section 5 demonstrates the numerical simulation results. Section 6 discusses the clinical aspect. The last section concludes some remarks.

## 2. Control (Treatment) Problem Definition

By using the state-space theory [8] for the modeling of dynamical systems, the state-space model of the system can be obtained to facilitate the control input (treatment) design for the system. Since the model of the system given in Equation (2) is a second-order differential equation, the state variables can be selected as follows:

(3) $$x\left(t\right)=\left[\begin{array}{c} x_{1}(t)\\ x_{2}(t) \end{array}\right]=\left[\begin{array}{c} x(t)\\ \dot{x}(t) \end{array}\right]$$

where x(t) is the state vector containing the state variables $x_{1}(t)$ and $x_{2}(t)$ that are selected as the mood, x(t), and the change rate of the mood, $\dot{x}\left(t\right)$. So the nonlinear state-space model of the system given in Equation (2) will be

(4) $$\begin{array}{c} {\dot{x}}_{1}\left(t\right)=x_{2}\left(t\right)\\ {\dot{x}}_{2}\left(t\right)=-cx_{1}\left(t\right)+bax_{2}\left(t\right)-bx_{1}^{2}(t)x_{2}(t)+u(t) \end{array}$$

which is the model to be utilized in the control input, $u\left(t\right)$, design. Note that the nonlinear state-space model given in Equation (4) models and justifies the nature of the illness; i.e., since the equilibrium points of the model is

(5) $$\left[\begin{array}{c} x_{1e}\\ x_{2e} \end{array}\right]=\left[\begin{array}{c} 0\\ 0 \end{array}\right]$$

the mood and its change rate, x(t) and $\dot{x}(t)$, respectively, converge to zero without any external perturbation and with zero initial conditions. Any values of initial conditions other than zero will lead to a mood swing.

By using the state-space model given in Equation (4), two control problems can be defined as follows: (i) drive the mood, x(t), to its equilibrium point (state), 0, using the control input signal, u(t), if all the system parameters, a, b, and c, are known; (ii) drive the mood, x(t), to its equilibrium point (state), 0, using the control input signal, u(t), even if all the system parameters, a, b, and c, are unknown. The former is defined as the exact model knowledge controller, and its design is given in following section. The latter is called an adaptive controller, and its design is provided in Section 4.

The main idea behind the treatment is to either decrease the amplitude of the oscillation, in other word, the mood swing, or completely quench it. So a controller (treatment) should also guarantee that the change rate of the mood, $x_{2}$, is also bounded. Since the control input signal, u(t), appears in the second state equation and the control objective is to stabilize both state variables, the Backstepping Technique [9] is a very useful tool for control design and is used in this study. For both controllers, Lyapunov-type arguments are used to design the controller and to prove the stability of the designed controllers. By using the MATLAB/Simulink environment [10], numerical simulation results are also provided to show the performance and feasibility of the proposed controllers. During the design of both controllers, the states, x(t) and $\dot{x}(t)$, are assumed to be measurable.

## 3. Exact Model Knowledge Controller

To apply the Backstepping procedure, let us add and subtract a virtual control input, $x_{2d}(t)$, to the right-hand side of the first state equation given in Equation (4), where

(6) $${\dot{x}}_{1}\left(t\right)=x_{2}\left(t\right)-x_{2d}(t)+x_{2d}(t)$$

The virtual control input, $x_{2d}$, is used to correlate the first and second state variables, $x_{1}(t)$ and $x_{2}(t)$, to design the control input signal, u(t), and stabilize $x_{1}(t)$. To end this step, we define the Backstepping variable as

(7) $$h(t)≜x_{2}\left(t\right)-x_{2d}(t)$$

and design the virtual control input signal as

(8) $$x_{2d}\left(t\right)=-{k_{1}x}_{1}(t)$$

where $k_{1}\in R^{+}$ is a positive control gain and allows the final dynamics of $x_{1}(t)$ to be expressed as

(9) $${\dot{x}}_{1}\left(t\right)=-k_{1}x_{1}\left(t\right)+h(t)$$

Investigating h(t) dynamics by Backstepping it yields

(10) $$\dot{h}\left(t\right)={\dot{x}}_{2}\left(t\right)-{\dot{x}}_{2d}(t)$$

and substituting $x_{2}$ dynamics from Equation (4) and $x_{2d}$ dynamics developed from Equation (8) into Equation (10) results in

(11) $$\dot{h}\left(t\right)=-cx_{1}\left(t\right)+bax_{2}\left(t\right)-bx_{1}^{2}x_{2}+u\left(t\right)+k_{1}^{2}x_{1}\left(t\right)-k_{1}h(t)$$

which can be expressed in a more compact form as

(12) $$\dot{h}\left(t\right)=W\theta +u\left(t\right)+k_{1}^{2}x_{1}\left(t\right)-k_{1}h(t)$$

where W is the regression vector with the elements of all known measurables represented as

(13) $$W≜\left[\begin{array}{ccc} -x_{1}(t) & x_{2}(t) & -x_{1}^{2}(t)x_{2}(t) \end{array}\right]$$

and θ is the parameter vector with elements of the system parameters, where

(14) $$\theta =\left[\begin{array}{c} c\\ ba\\ b \end{array}\right]$$

At that point, the control input signal can be designed as

(15) $$u\left(t\right)=-W\theta -K_{1}^{2}x_{1}(t)+K_{1}h(t)-x_{1}(t)-K_{2}h(t)$$

which allows the final dynamics for the Backstepping variable, h(t), to be expressed as

(16) $$\dot{h}(t)=-K_{2}h(t)-x_{1}(t)$$

where $K_{2}\in R^{+}$ is another control gain.

To analyze the effects of the exact model knowledge control input signals given in Equation (15) on the system’s stability, the following theorem and its proof can be used.

**Theorem** **1:** *For the system given in Equation (4), the control input signal defined in Equation (15) ensures that *$x_{1}(t)$ *converges to zero exponentially. Moreover, it guarantees the boundedness of all signals in the closed-loop system.*

**Proof of Theorem** **1:** To prove the theorem given above, we use the following nonnegative scalar function:

(17) $$V(t)=\frac{1}{2}x_{1}^{2}\left(t\right)+\frac{1}{2}h^{2}(t)$$

Each term of this function is always positive; namely, the function is positive definite. If two sides of (17) are differentiated with respect to time, where

(18) $$\dot{V}(t)=x_{1}(t){\dot{x}}_{1}(t)+h(t)\dot{h}(t)$$

and the expressions given in Equations (9) and (16) are substituted into Equation (18), the resulting equation will be

(19) $$\dot{V}(t)=-k_{1}x_{1}^{2}(t)-k_{2}h^{2}(t)$$

Considering Equations (17) and (19) together, it can be written that

(20) $$\dot{V}\left(t\right)=-\beta V(t)$$

where $\beta \in R^{+}$ is a positive constant, which has a solution like

(21) $$V\left(t\right)=V\left(0\right)exp\left\{-\beta t\right\}$$

where V(0) is the initial value of V(t) and $exp\left\{\cdot \right\}$ is the natural logarithm function. Equation (21) clearly says that V(t) goes to zero exponentially, while time goes to infinity. So its components, $x_{1}(t)$ and h(t), also converge to zero. This necessarily means that all signals in the closed-loop system are bounded, as claimed in Theorem 1. This ends the proof. □

## 4. Adaptive Controller

The exact model knowledge controller given in the previous section guarantees the boundedness of all signals in the closed-loop system as proved, but it needs the exact values of the system parameters, a, b, and c, to implement it. From a clinical point of view, determination of the exact values of these parameters dictates a personal identification process that may delay the treatment. Finding “exact” values of these parameters may also be unreasonable for some clinicians. For these reasons, an alternative controller may be needed to clinically implement the treatment without “exact knowledge” of these parameters. Adaptive control provides a useful tool for this aim.

The main idea behind the adaptive controller is to estimate the values of the unknown parameters by using the values of the measurable states. These estimations are not needed to converge their original values, as the key point is to be able to guarantee the boundedness of all signals despite the parameter uncertainties in the system model.

To design the adaptive controller, let us define

(22) $$\tilde{\theta }(t)=\theta -\hat{\theta }(t)$$

where $\hat{\theta }(t)$ is the dynamic time-varying estimation vector of the constant parameter vector, θ, and $\tilde{\theta }(t)$ is the estimation error vector. For the adaptive controller, the parameter vector, θ, given in Equation (14) cannot be used in designing the control input signal since it is assumed to be unknown. Instead, the dynamic time-varying estimation vector, $\hat{\theta }(t)$, can be used as long as this vector is continuously updated (adapted) using the measurable variables.

At that point, the adaptive control input signal can be redesigned as

(23) $$u\left(t\right)=-W\hat{\theta }-K_{1}^{2}x_{1}(t)+K_{1}h(t)-x_{1}(t)-K_{2}h(t)$$

which allows the final dynamics for the Backstepping variable, h(t), to be expressed as

(24) $$\dot{h}\left(t\right)=-K_{2}h\left(t\right)-x_{1}\left(t\right)-W\tilde{\theta }(t)$$

To analyze the effects of the exact model knowledge control input signals given in Equation (22) on the system’s stability, the following theorem and its proof can be used.

**Theorem** **2:** *For the system given in Equation (4), the control input signal defined in Equation (23) ensures that *$x_{1}(t)$ *converges to zero asymptotically while the elements of the parameter estimation error vector, *$\tilde{\theta }(t)$*, converge to some constants with a parameter update rule*

(25) $$\dot{\hat{\theta }}(t)=h(t)ГW^{T}(t)$$

*where* Г *is the three-by-three parameter update constant gain matrix. Moreover, it guarantees the boundedness of all signals in the closed-loop system.*

**Proof** **2:** To prove the theorem given above, we use the following nonnegative scalar function:

(26) $$V\left(t\right)=\frac{1}{2}x_{1}^{2}\left(t\right)+\frac{1}{2}h^{2}\left(t\right)+\frac{1}{2}{\tilde{\theta }}^{T}(t){Г}^{-1}\tilde{\theta }(t)$$

Each term of this function is always positive; namely, the function is positive definite. If two sides of (26) are differentiated with respect to time, where

(27) $$\dot{V}\left(t\right)=x_{1}\left(t\right){\dot{x}}_{1}\left(t\right)+h\left(t\right)\dot{h}\left(t\right)-{\tilde{\theta }}^{T}(t){Г}^{-1}\dot{\hat{\theta }}(t)$$

and the expressions given in Equations (23)–(25) are substituted into Equation (27), the resulting equation will be

(28) $$\dot{V}(t)=-k_{1}x_{1}^{2}(t)-k_{2}h^{2}(t)$$

Considering Equations (26) and (28) together, it is said that $x_{1}\left(t\right)$ and h(t) approach zero asymptotically while the elements of $\tilde{\theta }$ approach a constant since they do not appear in $\dot{V}$. This necessarily means that all signals in the closed-loop system are bounded, as claimed in Theorem 2. This ends the proof. □

A numerical simulation of the designed exact model knowledge controller is given in the following section.

## 5. Numerical Simulation

To show the performance and feasibility of the proposed treatment algorithms, numerical simulations were performed using the MATLAB/Simulink environment. For all the simulations, initial conditions were selected as $x_{1}\left(0\right)=0.5$ and $x_{2}\left(0\right)=0$. The parameter values were assigned as a=1, b=2, and c=9.

**Figure 2:**
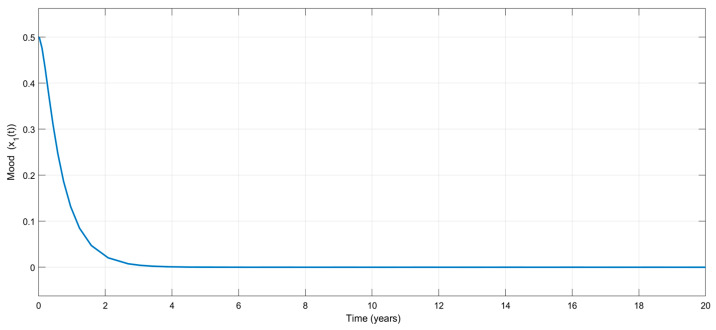
Time-dependent variation in the emotional mood, $x_{1}(t)$, for the exact model knowledge controller.

**Figure 3:**
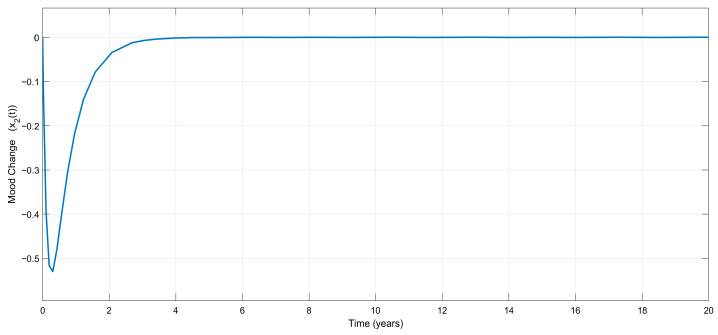
Time-dependent variation in the change rate of the emotional mood, $x_{2}(t)$, for the exact model knowledge controller.

For the exact model knowledge controller, the control gains were selected as $K_{1}=1.1$ and $K_{2}=10$. Figure 2 shows the time variation in the emotional mood, $x_{1}\left(t\right),$ for the exact model knowledge controller. Contrary to other available control treatment algorithms, the interesting part of the figure is that no mood change appears during the treatment, which is very attractive from a clinical point of view. Without any external disturbances, the treatment stabilizes mood, as shown in Figure 3, which visualizes the variation in the mood change, $x_{2}(t)$, with respect to time.

**Figure 4:**
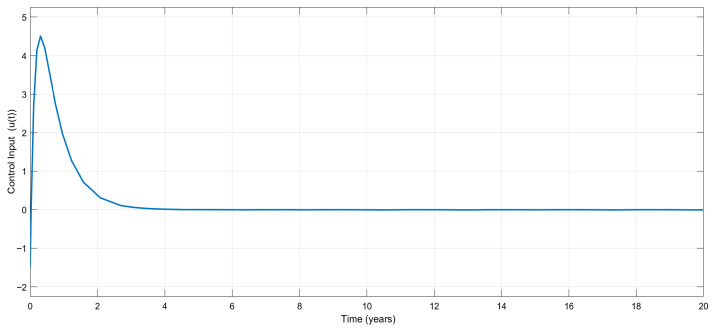
Time-dependent variation in the control input, u(t), for the exact model knowledge controller.

Figure 4 shows the variation in the control input signal, u(t), which is a combination of antidepressants and antipsychotics. As can be seen from the figure, a bounded and reasonable control effort is needed, and after a certain time period, treatment does not include antidepressants and antipsychotics; i.e., the control effort converges to zero. After that time, clinical treatment only includes the therapy dimension (if needed) until a certain external disturbance (if any) affects the patient.

Based on Figure 2, Figure 3 and Figure 4, it can be said that the numerical simulation results for the exact model knowledge controller are in good agreement with the theory given in Section 3.

For the adaptive controller, the control gains were selected as $K_{1}=1.22$ and $K_{2}=10$. Elements of the adaptation matrix were selected as

(29) $$Г=\left[\begin{array}{ccc} 0.1 & 0 & 0\\ 0 & 0.1 & 0\\ 0 & 0 & 0.1 \end{array}\right]$$

**Figure 5:**
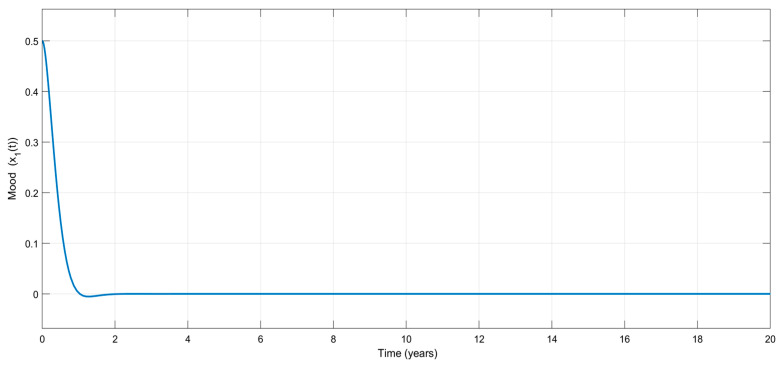
Time-dependent variation in the emotional mood, $x_{1}(t)$, for the adaptive controller.

**Figure 6:**
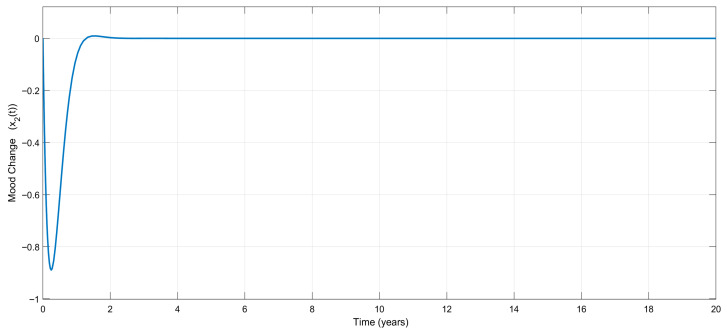
Time-dependent variation in the change rate of the emotional mood, $x_{2}(t)$, for the adaptive controller.

Figure 5 shows the time variation in the emotional mood, $x_{1}\left(t\right),$ for the adaptive controller. Once again, no considerable mood change appears during the treatment, which is very attractive from a clinical point of view. The treatment stabilizes mood, as shown in Figure 6, which visualizes the variation in the mood change, $x_{2}(t)$, with respect to time.

**Figure 7:**
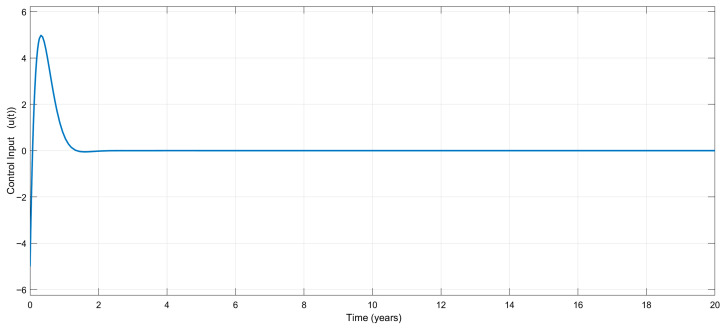
Time-dependent variation in the control input, u(t), for the adaptive controller.

Figure 7 shows the variation in the control input signal, u(t), which is bounded and reasonable, as claimed.

**Figure 8:**
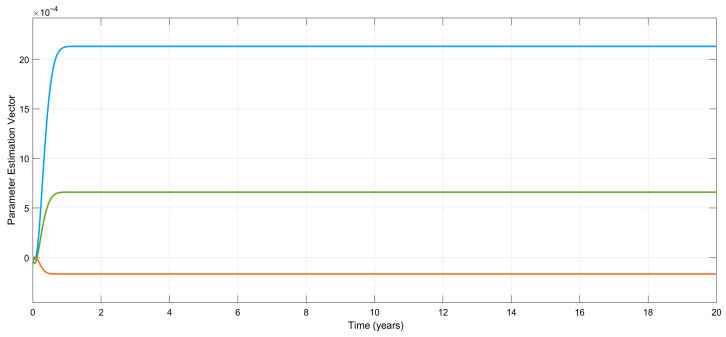
Time-dependent variation in the estimation errors of the uncertain parameters for the nonlinear adaptive controller.

Figure 8 shows the variations in the estimation errors of the uncertain parameters. As claimed, the estimations (and errors) converge to some constants, which necessarily means that the estimation errors also approach some constants. Even if they could not have been driven to zero, the control objective is still achieved; i.e., the emotional mood, $x_{1}(t)$, tends to its equilibrium point, 0, asymptotically in spite of the existence of uncertain parameters.

From Figure 5, Figure 6, Figure 7 and Figure 8, it can be said that the numerical simulation results for the adaptive controller are in good agreement with the theory given in Section 4.

## 6. Clinical Aspect

**Figure 9:**
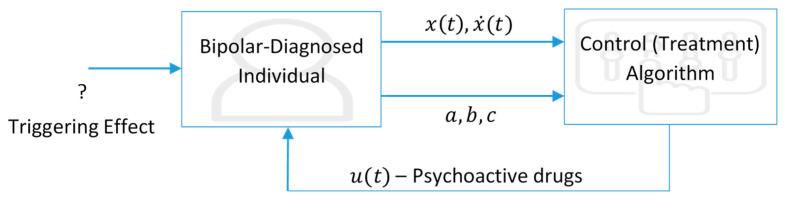
Schematic representation of the classical treatment process.

This chapter is designated to explain the clinical aspect and especially the clinical importance of the proposed treatment scheme. To do this, simply recall the two basic steps of the clinical efforts in this paper’s topic: (i) a diagnosis of the illness, and (ii) treatment of the illness. Even though the former is out of the scope of this study, one important bridge between the former and the latter one must be emphasized: characterization. By characterization, it is meant that the determination of the exact values of the system parameters *a*, *b*, and *c* is needed. Figure 9 shows a schematic representation of the treatment process starting from diagnosis.

**Figure 10:**
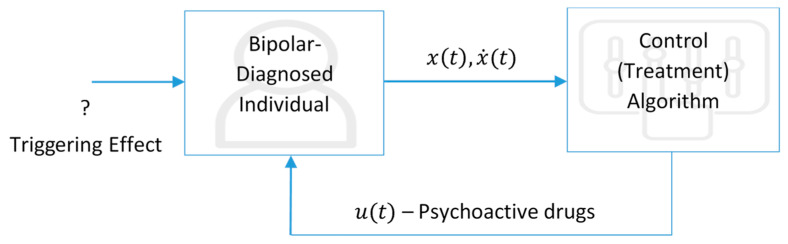
Reduced schematic representation of the classical treatment process.

The process starts with the diagnosis of Bipolar II Disorder triggered by any effect on the individual. After the diagnosis, a clinician should determine the values of both the mood and its change rate, as well as the parameters (a, b, and c) that are specific to each patient. The treatment algorithm proposed in this study is easily implemented using a software with a visual interface to produce the concentration (or dosage) of the psychoactive drugs as an output of the algorithm. This also allows any clinician who is not familiar with the math behind the algorithm to be able to apply the process. It is quite clear that the most challenging part of the process is to determine the exact values of the system parameters a, b, and c since no clinically evident mature methods are available to determine these parameters. One of the clinical advantages of this study is that it eliminates the need for determining the values of these parameters. The adaptive controller does not need the values of these parameters to stabilize the system. So the schematic diagram given in Figure 9 is reduced to the one in Figure 10, where no clinical effort is needed to determine the parameters.

Eliminating the need for determining the values of the parameters *a*, *b*, and *c* also consolidates the other advantage of the proposed treatment scheme: quenching the mood swing. As stated before, the goal of psychiatric treatment (usually psychoactive drugs) is to quench the oscillation completely, as proven analytically in Section 4 and numerically in Section 5, while the other studies available in the pertinent literature aim to substantially reduce the amplitude and frequency of the mood swing. The benefit the proposed algorithm offers is as follows: the quenching of a mood swing without the knowledge of patient-specific parameters.

After a certain period of treatment, relapse may occur, so a possible change in the values of these parameters should again be determined, and the proposed algorithm also eliminates this change to quench the oscillations. From a clinical view of the quenching effect, it is also important to state that the endogenous oscillations and the reactive components of mood can be separable, and the former has been successfully treated. It must be emphasized that the idea of treatment effects emerging only after treatment has concluded and of treatment being curative, with no further work needed, does not describe any psychotropic medications in existence. They all manage symptoms and stop working when they are discontinued, but they are reminiscent of skills-based cognitive-behavioral therapy for depression and panic disorder. Here, the patient can continue to improve after therapy ends as they gain fluency in their new skills, and the disorder may be cured.

Another important clinical aspect of the proposed treatment scheme is that it allows the clinician, after a certain period of time, to observe and record the steady-state values of the estimated parameters *a*, *b*, and *c*. Having the estimated values of these parameters allows the clinicians to utilize these values for some other treatment purposes, for example, for any case of relapse. Note that the vector of the parameters given by Equation (14) is

(30) $$\theta =\left[\begin{array}{c} c\\ ba\\ b \end{array}\right]$$

and the values of the parameters used in the simulation are assigned as a=1, b=2, and c=9. This necessarily means that the vector given in Equation (30) will be

(31) $$\theta =\left[\begin{array}{c} 9\\ 2\\ 2 \end{array}\right]$$

**Figure 11:**
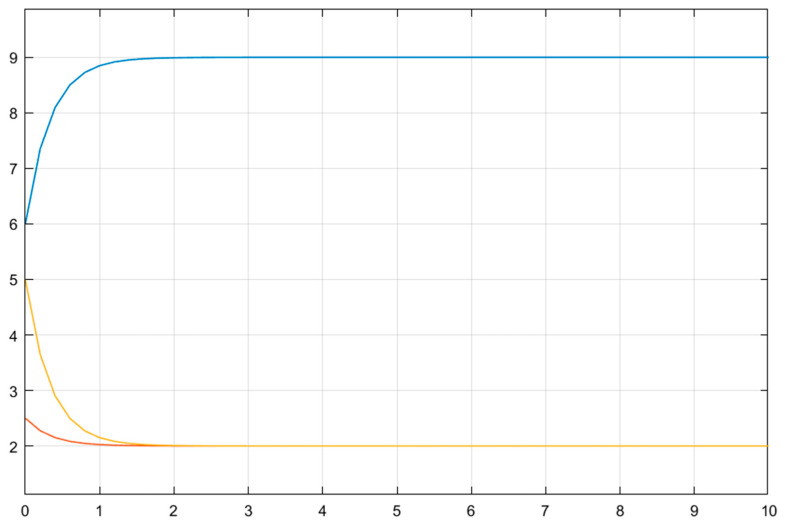
Time-dependent variation in the estimation of the uncertain parameters.

As can be seen from Figure 8, estimation errors converge to some small and ignorable values. If any clinician inputs their initial (estimated) values quite far from their steady-state values, the software will again output their converged estimation values. Figure 11 shows that if the initial conditions of the parameter estimations are selected as a=0.5, b=5, and c=6, the estimation values will again converge to a constant, as shown in Equation (31).

## 7. Conclusions

A nonlinear dynamical model of bipolar disorder has been used to characterize its nature. Two treatment (control) algorithms have been presented. Compared to the pertinent available literature on treatment approaches [11,12,13,14,15,16,17,18,19,20,21], two basic novelties have been proposed. First, it has been shown that a nonlinear exact model knowledge controller is capable of driving the emotional mood to its equilibrium point, zero, by quenching the mood swing not only at steady state but also during the transient period; i.e., no mood change, the ultimate threat, has been observed during the treatment in numerical simulations.

Secondly, it has also been shown that a nonlinear adaptive controller is capable of performing the same function despite the parameters of the model being uncertain, which is clinically more reasonable. The controller again drives the emotional mood to its equilibrium point, zero, by quenching the mood swing not only at steady state but also during the transient period even if all of the parameters in the nonlinear system model are assumed to be unknown.

Future remarks would be on the implementation of the proposed algorithms. The first step will be designing an observer to estimate the state variables, $x_{1}(t)$ and $x_{2}(t)$, that were assumed to be measurable in this study. Designing such an observer would facilitate the treatment for both clinicians and patients since it eliminates the physical measurement of mood and its change rate by using some physical indicators.

## Data Availability

The original contributions presented in this study are included in the article. Further inquiries can be directed to the corresponding author.
